# Supplementary material for: Resolving the full spectrum of human genome variation using Linked-Reads
Source: Genome Res. 2019 Apr;29(4):635–45. doi: 10.1101/gr.234443.118 (PMC6442396; doi:10.1101/gr.234443.118)
Supplement: Supplemental Material [file supp_gr.234443.118_Supplemental_longranger-2.2.zip › longranger-2.2.tar.bz2!/lr2.2-public/lib/go/src/code.google.com/vitess/vitess.io/_includes/page-meta.html]

{% if page.date %}

Updated {% if page.modified %}{{ page.modified | date: "%B %d, %Y" }}{% else %}{{ page.date | date: "%B %d, %Y" }}

{% endif %}

{% endif %}
